# Disulfidptosis, Hydrogen Sulfide, and Acute Kidney Injury: Emerging Mechanisms and Therapeutic Intersections

**DOI:** 10.3390/biom16101389

**Published:** 2026-09-23

**Authors:** Shreyasi Gupta, Subhadeep Mandal, Kalyan Banerjee, Anwita Bhattacharya, Robert E. Brainard, Utpal Sen

**Affiliations:** 1 Department of Zoology, Trivenidevi Bhalotia College, College Para Rd, Raniganj 713347, West Bengal, India; guptashreyasi2009@gmail.com (S.G.);; 2 Department of Physiology, University of Louisville School of Medicine, Louisville, KY 40202, USA

**Keywords:** disulfidptosis, H_2_S, acute kidney injury, cystine/glutamate antiporter

## Abstract

Acute kidney injury (AKI), a sudden and rapid loss of renal function that is often reversible, encompasses metabolic dysfunction, oxidative stress, inflammation, cytoskeletal collapse, and finally, renal cell death. Besides various types of cell death, like apoptosis, pyroptosis, necroptosis, and ferroptosis, disulfidptosis could be associated with AKI. Disulfidptosis is a novel form of regulated cell death driven by aberrant disulfide stress, which is triggered in glucose-starved cells exhibiting high expression of SLC7A11 (Solute Carrier Family 7 Member 11), which is a cystine/glutamate antiporter. Additionally, disulfidptosis is characterized by a significant disruption in the NADPH/NADP^+^ ratio and the unusual buildup of disulfides such as cystine (C_3_H_7_NO_2_ S). These alterations ultimately destabilize the F-actin network, leading to cell death. Hydrogen sulfide (H_2_S), a recognized gasotransmitter, serves as a cytoprotective, redox-regulating, and anti-inflammatory molecule that has attracted interest as a potential regulator of cellular redox equilibrium, actin behavior, and mitochondrial activity in the kidneys. This review aims to incorporate recent findings on the interfaces of disulfidptosis and H_2_S biology with AKI pathophysiology and potential therapeutic applications.

## 1. Introduction

Acute Kidney Injury (AKI) affects approximately 13.3 million people worldwide each year [1,2]. Despite significant research, the molecular mechanisms underlying AKI remain incompletely understood, highlighting the need for improved strategies for early diagnosis. In the pathophysiology of AKI, apoptosis and pyroptosis are two primary forms of cell death, and their presence and interplay significantly influence the progression of kidney injury [3,4,5]. AKI leads to direct mitochondrial DNA damage and excessive production of reactive oxygen species (ROS), resulting in mitochondrial dysfunction and the initiation of apoptosis. Activated caspase-3, a cysteine-aspartic protease, is involved in mediating DNA repair failure, degradation of the cytoskeleton, and the formation of apoptotic bodies [6]. Both stress-induced and programmed cell death are tightly regulated, sequential processes directed by intricate molecular cascades involving a variety of proteins, enzymes, hormones, and cytokines [7,8].

Several distinct modes of cell death have been identified and thoroughly investigated to date, including apoptosis, pyroptosis, necroptosis, ferroptosis, and others [9,10]. Increased cleaved Caspase-3 levels in cisplatin-induced kidney injury models confirm the activation of apoptosis [6]. Pyroptosis has recently been recognized as a key inflammatory form of cell death implicated in the development of AKI. This process involves the release of pro-inflammatory intracellular contents, primarily mediated by the gasdermin protein family [6,11]. In AKI, activation of pyroptosis pathways worsens tubular damage and promotes inflammation, thereby contributing to disease pathogenesis. Additional evidence highlights the role of Gasdermin E (GSDME)-mediated pyroptosis in various kidney injuries [12,13]. Notably, apoptosis and pyroptosis are not mutually exclusive; cells may transition between these two forms of cell death in response to different stimuli [6]. Therefore, a deeper understanding of programmed cell death may enhance our knowledge of disease mechanisms and open up novel therapeutic approaches for certain conditions.

Recently, disulfidptosis has been reported as a novel type of disulfide stress-mediated cell death [14,15,16]. Studies have shown that disulfidptosis occurs in cells with elevated levels of solute carrier family 7 member 11 (SLC7A11), a transporter responsible for exchanging cystine and glutamate during glucose starvation [17]. SLC7A11 expression increases in response to disulfide stress—a state of cellular oxidative stress caused by an excess of disulfide-bonded proteins. Disulfidptosis is characterized by the depletion of NADPH, resulting in an altered NADPH/NADP^+^ ratio, and the abnormal buildup of disulfides such as cystine. These disruptions ultimately lead to cell death through the collapse of the filamentous actin (F-actin) network [15,18,19]. In AKI, especially in diabetic nephropathy (DN), hyperglycemia leads to high SLC7A11 (cystine-glutamate antiporter) expression [20,21]. Under high-glucose-induced stress or energy failure, i.e., glucose starvation, SLC7A11 continues to import cystine, leading to its accumulation. Studies have identified disulfidptosis-related genes, such as *CXCL6*, *CD48*, *C1QB*, and *COL6A3*, which are significantly associated with diabetic nephropathy and a decline in the estimated glomerular filtration rate (eGFR) [22]. Inhibition of thioredoxin-interacting protein (TXNIP), a key regulator of disulfide stress, has been demonstrated to decrease kidney injury and enhance renal outcomes in diabetic models [23].

H_2_S, recognized as the third endogenous signaling molecule after carbon monoxide (CO) and nitric oxide (NO), plays a vital physiological role in mammals. Within the renal system, its synthesis is primarily catalyzed by cystathionine-γ-lyase (CSE), cystathionine-β-synthase (CBS), and 3-mercaptopyruvate sulfurtransferase (3-MST) [24]. Around 75% of renal cells can express H_2_S-synthesizing enzymes, and all known H_2_S biosynthetic pathways are present in the kidney, highlighting H_2_S’s integral role in the regulation of renal physiological processes [25]. Reduced endogenous H_2_S production has been found to aggravate kidney injury [26,27,28], while supplementation with exogenous H_2_S has been shown to reverse or mitigate the development and progression of various experimental AKI [29]. H_2_S generally acts as a protective, anti-apoptotic agent against kidney cell death, particularly in acute kidney injury (AKI), ischemic, and diabetic models [30].

In connection with H_2_S [31] and disulfidoptosis [32], cysteine (the reduced form of cystine) is an important molecule, as it is required for the synthesis of H_2_S, and its over-accumulation triggers disulfidoptosis. H_2_S can both induce and reduce disulfidptosis, depending on its concentration and the cellular environment, and whether it is produced endogenously or administered as a therapeutic donor [33]. High H_2_S doses, along with glucose starvation, trigger cytoskeletal collapse [34], whereas under physiological or protective conditions, low-dose release of H_2_S in the extracellular space induces a reduction of cystine to cysteine, increasing the amount of cysteine available as a substrate for glutathione (GSH) synthesis and enhancing cystine transport, which boosts GSH- and NADPH-dependent defenses [35], indicating attenuation of disulfidptosis. In this review, we discuss molecular mechanisms of disulfidptosis in connection with acute kidney injury and focus on the potential therapeutic applications of H_2_S in reducing disulfidptosis in AKI.

## 2. Disulfidptosis: An Emerging Mechanism of Regulated Cell Death

### 2.1. Molecular Basis of Disulfidptosis

Disulfidptosis is a relatively new concept of regulated cell death (RCD) distinct from apoptosis or ferroptosis [15,19,36,37,38,39], and its molecular mechanism is schematically represented in Figure 1.

It occurs when cells with high SLC7A11 (xCT) expression import excess cystine and exhaust their NADPH pool [32,34]. In response, the pentose phosphate pathway (PPP) readily provides NADPH to reduce cystine to cysteine. In contrast, when glucose is scarce or PPP is inhibited, NADPH levels fall, and imported cystine accumulates within cells [14,40,41], finally resulting in redox imbalance that causes aberrant formation of intermolecular disulfide bonds in actin and associated cytoskeletal proteins [14,39,41], which can lead to rapid F-actin network collapse and cell death—a pathway termed disulfidptosis [34,36,39,42,43]. In short, SLC7A11 overexpression and high cystine uptake (triggering NADPH depletion) initiate a cascade of disulfide bond formation in actin filaments, culminating in cytoskeletal “contraction” and disulfidoptotic death [14,18,41]. Several molecular regulators modulate this pathway. Key initiators include SLC7A11 (xCT) itself, which mediates cystine import; high SLC7A11 expression is required for disulfidptosis (genetic ablation of SLC7A11 prevents this death) [32,34]. Excess intracellular cystine drives NADPH consumption until depletion, directly invoking disulfide stress [32,34]. These stress-formed disulfide bridges in actin filaments cause disruption in cellular integrity by detaching F-actin from the membrane, often after a transient lamellipodial contraction [14,39,42,44].

It is established that F-actin collapse in disulfidptosis is facilitated by actin nucleators. Other actin-binding factors (e.g., cofilin and tropomyosin isoforms) likely modulate sensitivity by altering filament dynamics, though their roles are only beginning to be explored. Metabolic factors that alter the NADPH pool are also critical: for example, blocking glucose uptake or the PPP (e.g., 2-deoxyglucose) supplies glutathione-reducing equivalents to prevent disulfidptosis [14]. Conversely, inhibition of NADPH-generating enzymes (e.g., Nicotinamide Adenine Dinucleotide Kinase (NADK) or glucose-6-phosphate dehydrogenase) or reducing systems (e.g., thioredoxin reductase 1) makes cells more susceptible [14,45]. Disulfidptosis is regulated by several factors, which are listed in Table 1.

**Figure 1:**
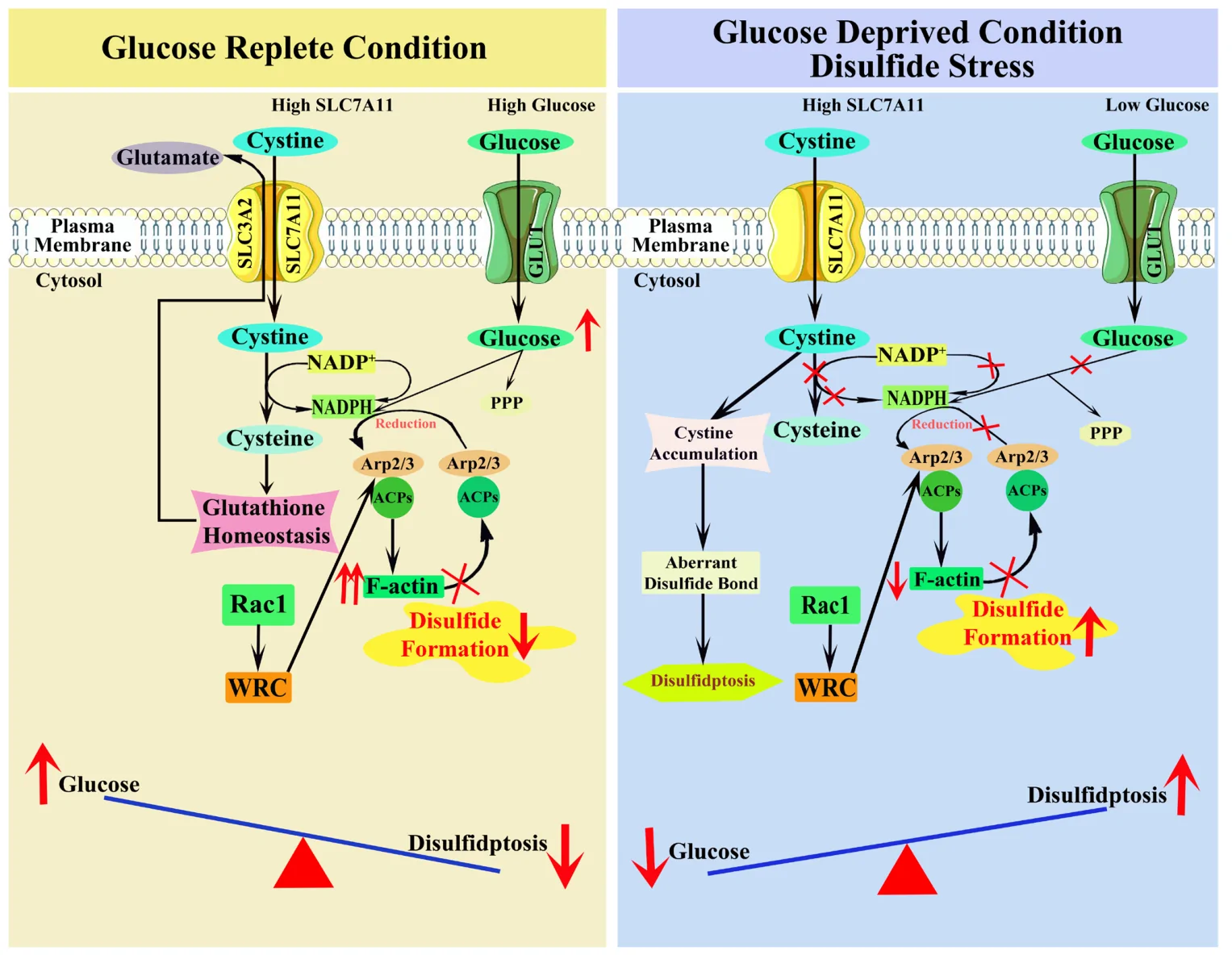
An integrated schematic illustrates the molecular mechanism underlying disulfidptosis. Glucose uptake activates the pentose phosphate pathway (PPP), resulting in the production of NADPH. NADPH supplies the reducing power necessary for converting cystine, imported via the SLC7A11 transmembrane amino acid transporter (which functions as a cystine/glutamate antiporter), into cysteine. When glucose is abundant (**left panel**), sufficient NADPH maintains cellular redox balance, prevents abnormal disulfide bond formation in actin cytoskeletal proteins (ACPs), preserves F-actin structure, and supports cell survival. In contrast, under disulfide stress conditions, such as glucose deprivation or PPP inhibition in cells with high SLC7A11 expression (**right panel**), NADPH levels fall [46]. This impairs cystine reduction, leading to intracellular accumulation of cystine and other disulfide-containing metabolites, and promotes excessive disulfide bond formation within ACPs [47]. The result is oxidation and collapse of the F-actin network, destabilization of the cytoskeleton, and the onset of disulfidptosis. The Rac1–WRC–Arp2/3 (WRC: WAVE Regulatory Complex) signaling pathway enhances branched actin polymerization and lamellipodia formation, creating a structural environment conducive to pathological disulfide bond formation in actin filaments and intensifying disulfidptotic cell death [48]. Whether disulfide stress also triggers widespread disulfide bonding in other actin-based cellular structures beyond lamellipodia remains to be determined. Red color ↑ indicate increase, ↓ indicate decrease, and red color X represents blockade.

**Table 1:** The key triggers, regulators, and hallmarks of disulfidptosis are summarized below.

Component	Role/Category	Function or Marker	References
SLC7A11 (xCT)	Cystine transporter (Trigger/Regulator)	Imports cystine; overexpression drives NADPH depletion and disulfidptosis	[32,34]
Extracellular cysteine	Metabolic trigger	Elevated uptake consumes NADPH, increases intracellular disulfides	[14,37,49]
Glucose starvation/GLUT inhibition	Metabolic trigger	Limits NADPH supply; required co-trigger with SLC7A11	[32]
NADPH pool (PPP, NADK, TrxR1)	Redox regulator	NADPH depletion is central; PPP inhibition or TrxR1 inhibition triggers death	[14,45]
Rac1	Actin polymerization regulator (Regulator)	Promotes lamellipodia; its activation enhances disulfidptosis	[14,15,19,37,39,45,50]
WAVE regulatory complex (NCK-associated protein 1 (NCKAP1)/Cytoplasmic FMR1-interacting protein 1 (CYFIP1), Arp2/3)	Actin nucleation (Regulator)	Drives branched F-actin formation; required for disulfide-bond network; its loss inhibits disulfidptosis	[14,32,34,39,51]
Cytoskeletal proteins (e.g., actin, cofilin)	Effector/Target	Form aberrant intermolecular disulfide bonds under stress; actin collapse is hallmark of death	[14,44]
Disulfide bond reducers (DTT: Dithiothreitol; 2ME: 2-mercaptoethanol)	Inhibitors/Markers	Rescue cells by breaking abnormal disulfides (diagnostic of pathway)	[14,45]
Markers	Morphology/biochemical changes	F-actin network collapse; ↑NADP^+^/NADPH ratio; cytosolic cystine/disulfide accumulation	[14,32]

### 2.2. Sulfide Metabolism, Disulfidptosis, and Ferroptosis

Sulfide metabolism links the interconnected pathways of cellular antioxidant defense and regulated cell death, particularly through the SLC7A11 cystine/glutamate antiporter [52]. While normal sulfide flux processes cysteine to generate H_2_S or glutathione, which glutathione peroxidase 4 (GPX4) uses to prevent iron-dependent, lipid-peroxidative ferroptosis, glucose starvation and high SLC7A11 activity instead deplete NADPH, causing cystine overload, aberrant disulfide cross-linking of actin cytoskeletal proteins, and cell death via disulfidptosis [52].

Cysteine acts as a key node in intracellular sulfur metabolism, connecting H_2_S production to redox-sensitive cell death pathways. Cysteine is metabolized to H_2_S through multiple enzymatic routes: (i) the transsulfuration pathway via CBS and CSE; and (ii) the mitochondrial pathway involving cysteine aminotransferase (CAT) and 3-mercaptopyruvate sulfurtransferase (3-MST), in which cysteine is converted to 3-mercaptopyruvate (3-MP) and subsequently to H_2_S. In addition, peroxisomal D-amino acid oxidase (DAO) also converts D-cysteine into 3-MP, which is then transported into mitochondria, where 3-mercaptopyruvate sulfurtransferase (3-MST) uses it to generate H_2_S. CSE can also catalyze H_2_S production from L-homocysteine. Concurrently, cysteine metabolism integrates with regulated cell death pathways. Accumulation of cystine and impaired NADPH-dependent reduction promote aberrant disulfide bond formation in cytoskeletal proteins, driving disulfidptosis. In contrast, cysteine-derived GSH supports ferroptosis resistance by enabling GPX4 to reduce lipid hydroperoxides (LOOH) to non-toxic lipid alcohols (LOH). Thus, sulfur metabolism coordinates H_2_S signaling, redox homeostasis, cytoskeletal integrity, and the balance between ferroptosis and disulfidptosis (Figure 2).

### 2.3. Disulfidptosis Relevance to Kidney Tissue

Renal tubular epithelial cells are susceptible to disulfidptosis under stress. Proximal tubules in particular have very high metabolic demand and rely on the pentose phosphate pathway to generate NADPH for antioxidant defense [39]. Ischemia–reperfusion injury in the kidney, e.g., severe hypoglycemia or oxidative stress, depletes glucose and NADPH, creating conditions that can trigger disulfidptosis [14,39,42,44]. Although baseline SLC7A11 levels in normal kidneys are modest, in hypoxic or toxic injury SLC7A11 is upregulated (via Nrf2/HIF-1α) in renal epithelial cells [45,53]. Hypoxia-inducible factor–1α (HIF-1α) directly regulates SLC7A11 in ischemic kidneys [53]. These findings underscore that cystine transport (via SLC7A11) and NADPH-dependent redox buffering are critical in tubular cells. Emerging evidence hints that redox-dependent regulated cell death contributes to AKI. Ferroptosis, also involving SLC7A11, is well documented in ischemic and toxic AKI [53,54,55], and interventions targeting SLC7A11/Nrf2 pathways affect renal outcome. Similarly, it can be proposed that in an ischemic proximal tubule with high cystine absorption but reduced NADPH production, disulfide stress and disulfidptosis could occur. For example, supplying NADPH precursors such as fructose-1,6-bisphosphate or enhancing reducing pathways has been protective in some AKI models—interventions that would also suppress disulfidptosis [14,53]. Direct studies of disulfidptosis in AKI are not yet reported, but the convergence of high SLC7A11 flux, intense redox demand, and episodes of glucose deprivation in tubular cells suggests this pathway could contribute to acute tubular injury. Understanding this possibility may offer new biomarkers or therapies, e.g., modulating SLC7A11 or NADPH sources for AKI.

## 3. H_2_S: Biology and Renal Functions

### 3.1. Endogenous Production of H_2_S and Signaling

Although H_2_S was once considered solely a toxic gas, it is now recognized as a gaseous signaling molecule, sharing certain similarities with NO and CO [56]. Unlike NO and CO, H_2_S is acidic, enabling it to dissolve readily in water. Additionally, due to its high lipophilicity, H_2_S can freely diffuse across the cell membranes of all cell types [57]. H_2_S is produced in mammalian cells by several enzymatic pathways [58]. The classic enzymes are CBS and CSE, which use homocysteine and cysteine in the transsulfuration pathway, and 3-mercaptopyruvate sulfurtransferase (3-MST), which works with CAT in mitochondria [59,60]. In brief: CBS and CSE in the cytosol convert homocysteine (with serine) to cystathionine and then to cysteine, ultimately generating H_2_S. CSE can also act directly on L-homocysteine to release H_2_S plus α-ketobutyrate. A parallel route converts L-cysteine to 3-mercaptopyruvate (3-MP) via CAT, and 3-MST then converts 3-MP to H_2_S in mitochondria [60,61]. A fourth pathway involves D-cysteine: peroxisomal D-amino acid oxidase generates 3-MP from D-cysteine, which is transported into mitochondria for 3-MST to produce H_2_S [60,61,62,63]. Notably, in the kidney, D-cysteine is a major substrate, yielding much more H_2_S than L-cysteine [62]. All three H_2_S-synthesizing enzymes (CBS, CSE, and 3-MST) are highly expressed in the renal cortex (especially proximal tubules); in many species, CSE is the dominant isoform in the kidney, often far more abundant than CBS [60].

Once generated, H_2_S signals mainly by *S*-sulfhydrating reactive cysteine residues on target proteins [60]. This post-translational modification (adding an –SSH group) can dramatically alter enzyme activity or protein interactions. For example, H_2_S affects the S-sulfuration of numerous proteins, including metabolic enzymes (e.g., GAPDH) and structural proteins (actin, tubulin) [64]. In endothelial and smooth muscle cells, *S*-sulfhydration of potassium channels causes membrane hyperpolarization and vasodilation [65]. Thus, H_2_S is considered an endothelium-derived hyperpolarizing factor that lowers vascular tone by activating K^+^ channels via sulfhydration [66].

Beyond direct sulfhydration, H_2_S exerts potent antioxidant effects. It can donate electrons within mitochondria to stimulate oxidative phosphorylation and ATP production under hypoxic conditions [67]. Concomitantly, H_2_S limits ROS in several ways. It reversibly inhibits NADPH oxidase and activates antioxidant defenses. For example, H_2_S S-sulfhydrates the Keap1 protein (at Cys151), causing stabilization and nuclear translocation of Nrf2 [60]. Nrf2 then upregulates genes for glutathione synthesis and other antioxidants, preserving glutathione redox status and mitigating oxidative injury [60]. Thus, H_2_S preserves cell redox balance (e.g., via glutathione) and mitochondrial integrity during stress [67].

Our bodies naturally generate H_2_S, a gas involved in numerous physiological processes such as regulating blood pressure, modulating inflammation, supporting immune responses, influencing energy metabolism, and maintaining vascular health, among other functions [68,69]. Research has shown that H_2_S possesses anti-aging properties [70], largely due to its antioxidant, anti-inflammatory, and cytoprotective effects [71,72,73]. Tissue damage from free radicals is considered a major factor in aging and the development of age-related diseases. H_2_S helps counteract oxidative stress and may slow the aging process by scavenging ROS and enhancing the activity of antioxidant enzymes [74,75]. H_2_S inhibits pro-inflammatory cytokines, which are proteins that trigger inflammation, thereby helping to reduce inflammatory responses [72].

Mitochondrial function naturally declines with age, contributing to various age-related disorders. H_2_S has been shown to enhance mitochondrial function by increasing ATP production [76,77]. Age-related effects can vary between tissues; for example, the cardiovascular system is more vulnerable to age-associated changes than the liver [78,79,80,81]. While structural and functional alterations occur in the kidney with aging, the mechanisms behind these changes remain poorly understood [82]. Given these age-related changes in the kidney, it is likely that H_2_S production and signaling are also diminished. Reduced endogenous H_2_S levels have been linked to the progression of atherosclerosis, an age-related disease [83]. Moreover, inhibition of CBS expression in human umbilical vein endothelial cells has been shown to induce premature cellular aging [84]. Aging may also lower H_2_S production by causing oxidative damage to the CBS and CSE enzymes [85].

In summary, endogenous H_2_S (produced chiefly by CBS, CSE, and 3-MST/CAT) signals through multiple routes: *S*-sulfhydration of protein thiols, activation of pro-survival transcription factors (like Nrf2), modulation of ion channels and enzymes, and direct antioxidant effects [27,60]. These mechanisms allow H_2_S to influence vascular tone, metabolism, and inflammation in physiological and pathophysiological contexts.

### 3.2. H_2_S in Kidney Physiology

H_2_S is essential for physiological homeostasis, acting through multiple pathways [86]. It modulates kinases, ion channels, and transcription factors via S-sulfhydration of cysteine residues. Additionally, H_2_S interacts with heme in heme-containing proteins, serves as a free radical scavenger, and donates electrons to the mitochondrial electron transport chain, thereby boosting mitochondrial ATP production and regulating cellular bioenergetics [27,64,87,88,89,90]. Notably, the actions of H_2_S vary by tissue type. In the following section, we concentrate on its specific functions within the kidney.

H_2_S serves important regulatory and protective functions in the kidney. Research has shown that H_2_S modulates cellular processes within the kidney through various mechanisms, resulting in multiple downstream effects. For example, intrarenal administration of H_2_S donors such as NaHS increases GFR, urinary sodium excretion (UNaV), and urinary potassium excretion (UKV) [91,92]. A similar effect is observed with L-cysteine infusion, a substrate for endogenous H_2_S production [91]. Conversely, inhibition of endogenous H_2_S synthesis using aminooxy acetic acid (AOAA, a CBS inhibitor) combined with DL-propargylglycine (PAG, a non-specific CSE inhibitor) reduces GFR, UNaV, and UKV, suggesting that H_2_S is essential for normal renal function [61]. Further studies indicate that CBS and CSE enzymes compensate for each other in the regulation of renal function, as inhibition of only one enzyme (either AOAA or PAG alone) does not significantly affect renal parameters [93,94].

H_2_S induces vasodilation and inhibits the activity of Na^+^/K^+^-ATPase (NKA) and the Na^+^-K^+^-Cl^−^-cotransporter (NKCC), resulting in increased renal blood flow (RBF), elevated GFR, and enhanced natriuresis (U_Na_V_2_) and kaliuresis (U_K_V) [94] (Figure 3). Despite these effects, the precise impact of H_2_S on the kidney’s ionic exchange mechanisms remains uncertain. Evidence indicates that H_2_S also plays a role in renal water regulation [87,90,94,95]. Specifically, H_2_S upregulates renal aquaporin 2 (AQP2) expression by activating the intracellular cyclic adenosine monophosphate (cAMP)/protein kinase A (PKA) signaling pathway [94,96]. This leads to improved urine concentration and reduced urine osmolality, particularly in disorders of water balance such as nephrogenic diabetes insipidus [94,95,96]. It also maintains renal blood flow and glomerular filtration by vasodilating renal vessels. H_2_S relaxes vascular smooth muscle via the same K^+^-channel hyperpolarization [60,65]. In intra-renal infusion studies, H_2_S donors (e.g., NaHS) markedly increase renal blood flow and sodium excretion; conversely, blockade of H_2_S synthesis reduces flow and GFR [60,65,67]. Mechanistically, this vasodilatory effect works in tandem with NO. H_2_S can inhibit phosphodiesterases and boost NO bioavailability, lowering renal vascular resistance and engaging afferent/efferent arteriole dilation [60,67]. By preserving perfusion, H_2_S ensures oxygen delivery to nephrons and optimizes tubular filtration under diverse conditions. H_2_S is essential for regulating blood pressure, reducing proteinuria, and slowing the progression of renal dysfunction and structural deterioration by inhibiting angiotensin II and enhancing NO production [97]. Consequently, H_2_S could emerge as a promising therapeutic target for hypertensive nephropathy.

The release of renin from juxtaglomerular (JG) cells determines RAS activity, a process modulated by intracellular cAMP [61,98,99]. The renin–angiotensin–aldosterone system (RAAS) is a complex endocrine network responsible for regulating electrolyte balance and blood volume, thereby maintaining blood pressure [100,101,102]. H_2_S has been found to lower cAMP levels by inhibiting adenylyl cyclase (AC) in various cell types, implying that it may influence renin release and decrease angiotensin II levels [61]. Ming et al. reported that administration of the H_2_S donor NaHS both prevented and reversed hypertension in the two-kidney, one-clip (2K1C) model of renovascular hypertension [61]. Conversely, in normotensive rats and those with a one-kidney, one-clip (1K1C) model—both exhibiting normal plasma renin activity—NaHS did not affect blood pressure or plasma renin activity [61,94]. Studies using primary cultures of renin-rich kidney cells further demonstrated that NaHS substantially reduced intracellular cAMP levels and suppressed renin activity [61,94]. Laggner et al. showed that H_2_S directly inhibits angiotensin-converting enzyme (ACE) activity in a dose-dependent manner [103]. Additionally, recent evidence indicates that both AC and ROS are targets of H_2_S, as its renin-lowering effects in diabetic nephropathy (DN) suggest the involvement of multiple mechanisms [61,94].

Beyond hemodynamics, H_2_S suppresses renal inflammation. It inhibits leukocyte–endothelial interactions and blunts pro-inflammatory cytokine production. In rodent models, H_2_S donors markedly reduce leukocyte adherence to the microvascular endothelium and neutrophil infiltration [71,104,105]. H_2_S achieves this in part via K_ATP_ channel activation on endothelial cells, which prevents adhesion molecule expression [71,106]. In renal disease models, H_2_S lowers key cytokines: for example, in lipopolysaccharide (LPS)-induced AKI, H_2_S treatment attenuates the surge of TNF-α and IL-1β in kidney tissue [107]. Conversely, H_2_S deficiency (both genetic or pharmacologic) worsens renal inflammation. These anti-inflammatory actions likely involve H_2_S-sulfhydration of NF-κB and reduced activation of inflammasomes. Overall, H_2_S stabilizes the renal immune milieu—inhibiting leukocyte adhesion, cytokine release, and edema—thereby protecting tubules and glomeruli from immune-mediated injury [71,107].

At the cellular level, H_2_S optimizes tubular cell metabolism and survival. It serves as a mitochondrial electron donor, enhancing ATP production, especially under hypoxia [67]. H_2_S can improve the efficiency of oxidative phosphorylation in proximal tubule mitochondria, compensating for oxygen deficits during ischemia. At the same time, its antioxidant signaling (via Nrf2 and other pathways) preserves mitochondrial and endoplasmic reticulum (ER) function [60,67]. For example, during ischemia–reperfusion injury, H_2_S suppresses ROS accumulation and prevents calcium overload in tubular cells [60,67]. H_2_S also directly attenuates ER stress: it S-sulfhydrates ER-located Protein Tyrosine Phosphatase 1B (PTP1B) to modulate the unfolded protein response [60]. These effects collectively reduce tubular oxidative stress, protein misfolding, and apoptosis.

Emerging evidence also highlights that H_2_S regulates autophagy and mitophagy in renal epithelium. Autophagy is a cellular self-recycling process vital for clearing damaged proteins and mitochondria, especially in long-lived tubular cells. Studies show that H_2_S donors enhance autophagic flux in kidney cells during injury, which correlates with less inflammation and apoptosis [108]. In septic AKI models, exogenous H_2_S boosts autophagosome formation (detected by Microtubule-Associated Protein 1 Light Chain 3-II (LC3-II) increase) in tubular cells and reduces IL-1β release [108]. Blocking autophagy (e.g., with 3-MA; 3-Methyladenine) reverses much of H_2_S’s protection in AKI, suggesting that H_2_S-mediated induction of autophagy/mitophagy is essential for tubule repair [108]. Thus, by promoting appropriate autophagy and removal of damaged mitochondria, H_2_S helps maintain tubular homeostasis and aids recovery after injury.

H_2_S acts as an oxygen sensor in the renal medulla under hypoxic conditions, where its production increases to help restore oxygen levels. This rise in H_2_S promotes elevated renal blood flow (RBF) and GFR while also inhibiting tubular transit [94]. Although H_2_S can be synthesized without oxygen, its breakdown via mitochondrial oxidative metabolism is oxygen-dependent [109]. Because the renal medulla has lower oxygen availability than the cortex, H_2_S accumulates more in this region [110]. The resulting hypoxic environment, due to low oxygen partial pressure, leads to further H_2_S buildup. This accumulation enhances H_2_S-mediated activities, such as electron donation for ATP synthesis within mitochondria, and supports oxygen homeostasis by boosting medullary blood flow and reducing tubular sodium transport—responsible for about 60% of renal oxygen consumption [93,94,109,110,111,112,113,114] (Figure 3). Additionally, under hypoxia, CBS and CSE may translocate to mitochondria and stimulate H_2_S production [89,94,115]. However, further research is necessary to clarify the precise mechanisms and downstream signaling pathways involved.

Clinically, dysregulation of H_2_S signaling accompanies AKI. In ischemia–reperfusion models, expression of CSE/CBS falls, and H_2_S levels drop in the kidney; H_2_S supplementation improves perfusion and tubular recovery [60,67]. Similarly, in toxin-induced nephropathies (e.g., cisplatin injury) and sepsis-associated AKI, H_2_S bioavailability is reduced [60,107]. For instance, AKI patients have elevated serum creatinine, TNF-α and IL-1β but significantly lower circulating H_2_S compared to controls [107]. In LPS-induced (septic) AKI, renal 3-MST activity and H_2_S levels decline sharply, correlating with worse kidney function [107]. Restoring H_2_S (e.g., with donors) in these settings attenuates inflammation and oxidative damage and promotes autophagic repair [107,108]. Thus, loss of endogenous H_2_S (impaired production or increased clearance) contributes to AKI pathogenesis, while therapeutic H_2_S can mitigate injury.

## 4. Intersection Between Disulfidptosis and H_2_S in AKI

Disulfidptosis is driven by intracellular redox failure: excessive cystine uptake (via SLC7A11) under glucose/NADPH limitation leads to buildup of oxidized disulfide bonds (e.g., in actin) and cytoskeletal collapse [14,33,116,117]. In contrast, H_2_S is a gaseous reductant that modulates thiol chemistry. H_2_S (as HS^−^) reacts with oxidized thiols—converting protein disulfides or sulfenic acids into persulfides [118]. Thus, H_2_S can “rescue” proteins from excessive disulfide bonding. In kidneys, endogenous H_2_S is often depleted in AKI, and exogenous H_2_S donors (e.g., NaHS) improve renal function by reducing oxidative stress and inflammation [107]. For example, NaHS administration in septic (LPS-induced) AKI lowers creatinine/BUN and suppresses TLR4/NLRP3-mediated inflammation and lipid peroxidation [107]. Similarly, H_2_S donors protect against cisplatin nephrotoxicity by limiting tubular cell death [86].

These pathways converge on thiol–disulfide redox homeostasis. Disulfidptosis begins when NADPH levels are depleted, hindering the reduction of imported cystine. This results in the buildup of intracellular disulfides and causes cytoskeletal collapse during disulfide stress [15]. In contrast, H_2_S enhances cellular reducing capacity through multiple mechanisms. H_2_S directly converts protein disulfides to persulfides and promotes protein persulfidation, thereby protecting reactive cysteine residues from irreversible oxidation Importantly, H_2_S persulfidates the NADPH oxidase subunit p47^phox^, suppressing NADPH oxidase activity and attenuating ROS production [119]. Concurrently, H_2_S activates AKT signaling and promotes Keap1 persulfidation, resulting in Nrf2 stabilization and transcriptional upregulation of antioxidant genes such as Heme Oxygenase-1 (HO-1) and NAD(P)H:quinone dehydrogenase 1 (NQO1) [120,121,122].

Collectively, both disulfidptosis and H_2_S signaling are fundamentally governed by thiol–disulfide equilibrium: disulfidptosis is driven by excessive cystine-derived disulfide accumulation under reductive failure, whereas H_2_S donors restore redox balance by replenishing reduced thiols and activating endogenous antioxidant defenses. In AKI, a condition characterized by profound oxidative and metabolic stress [123,124], therapeutic augmentation of H_2_S signaling and intracellular thiol pools may counteract NADPH depletion and mitigate the molecular triggers of disulfidptosis.

### 4.1. Redox Imbalance as a Common Nexus of AKI, H_2_S and Disulfidptosis

AKI is marked by abrupt tubular damage driven by redox imbalance, high ROS and depleted NADPH, which tips thiol/disulfide chemistry toward oxidation. In this context, two emerging pathways, disulfidptosis (a novel cell death mode) and H_2_S signaling, both pivot on thiol/disulfide redox [64,125]. Disulfidptosis is triggered by an intracellular NADPH shortage that allows excessive disulfide (S–S) bond formation in proteins, whereas H_2_S, a biologic reductant, donates sulfur to form persulfides (–SSH) on protein cysteines (S-sulfhydration), enhancing antioxidant defenses. Intriguingly, their effects on the actin cytoskeleton are opposite: disulfidptosis induces aberrant actin crosslinking and collapse, while H_2_S-mediated sulfhydration stabilizes actin and promotes polymerization [60,126]. Thus, in AKI, where NADPH is often low and ROS high, disulfidptosis favoring disulfide stress may compete with H_2_S-driven thiol preservation. In effect, disulfidptosis accelerates tubular cell death under oxidative pressure, whereas H_2_S boosts thiol reduction and protects tubules. In this review, we discuss how these pathways intersect in AKI, both hinging on thiol/disulfide chemistry and redox balance.

Disulfidptosis is a recently discovered form of regulated cell death distinct from apoptosis, ferroptosis, and other forms of cell death. It was first described in 2023 in cells with high SLC7A11 expression (a cystine–glutamate antiporter) subjected to glucose starvation [14,34,126]. Under normal conditions, NADPH from the pentose phosphate pathway keeps cystine reduced to cysteine. But when NADPH is depleted (e.g., by glucose deprivation) while cystine imports remain high, reducing power is lost. The result is disulfide stress: intracellular cystine and other thiols form excess S–S bonds. In particular, abundant cytoskeletal proteins (actin) acquire aberrant disulfide crosslinks, causing the F-actin network to contract catastrophically [126]. Cells “survive if the reduced form of NADPH counteracts disulfides,” but disulfidptosis occurs when the balance breaks down [126]. Experimental evidence confirms that boosting reducing agents (e.g., β-mercaptoethanol) suppress this death, whereas oxidizing agents exacerbate it [14]. In other words, disulfidptosis is fundamentally a thiol–disulfide redox catastrophe driven by NADPH depletion.

Mechanistically, SLC7A11 high cells import large amounts of cystine, which must be reduced at the expense of NADPH. When glucose supply (and thus NADPH generation) is insufficient, NADPH/NADP+ drops dramatically. Liu et al. showed that under such stress, cells exhibit a ~1.5-fold increase in protein disulfide bonds and massive formation of mixed disulfides involving actin-binding proteins [14,126]. Fluorescence microscopy revealed striking cell shrinkage and F-actin contraction in starved cells [14,126]. Recent computational studies identified a connection between disulfidptosis-related genes and diabetic nephropathy and hypothesized that inhibition of disulfidptosis might delay kidney function decline [22]. Although direct studies of disulfidptosis in AKI are scarce, AKI is known to deplete NADPH and surge ROS (e.g., ischemia/reperfusion), conditions which in turn induce disulfide stress [34,60].

H_2_S is an endogenous gasotransmitter produced abundantly in the kidneys [60,127]. It is synthesized from cysteine via enzymes (CBS, CSE, 3-MST) in multiple pathways (including a kidney-specific D-cysteine/DAO–3MST route) [27]. Unlike disulfidptosis, H_2_S strengthens the thiol redox network. H_2_S reacts with cysteine thiols on proteins to form persulfides (–SSH). Mustafa et al. first showed that 10–25% of many cellular proteins are basally sulfhydrated, including actin, tubulin, and GAPDH [64,65]. Critically, sulfhydration generally enhances protein activity or stability. For actin, H_2_S-driven persulfidation promotes actin polymerization and cytoskeletal assembly [64]. In experimental systems, H_2_S donors (e.g., NaHS) boost actin polymerization, whereas blocking sulfhydration (with reducing agents) reverses this effect. Thus, H_2_S preserves a dynamic, polymerized actin network, in contrast to the crosslinked contraction seen in disulfidptosis. Beyond actin, H_2_S exerts broad antioxidant effects. It enhances the intracellular reducing capacity in multiple ways: by increasing GSH synthesis (through homocysteine transport and cysteine availability), by activating antioxidant enzymes (e.g., superoxide dismutase) and by inhibiting NADPH oxidases that generate ROS [128,129]. H_2_S also counters the very drivers of disulfide stress. It also modulates redox signaling: sulfhydration of proteins like p66Shc (at Cys59) blocks mitochondrial ROS production [130], and sulfhydration of Keap1 at Cys151 frees Nrf2 to induce antioxidant genes [120]. Moreover, H_2_S has anti-inflammatory and vasodilatory roles in AKI models, but the redox actions appear central. Notably, genetic or pharmacologic depletion of H_2_S (e.g., inhibiting CSE) worsens AKI, whereas H_2_S donors (NaHS, GYY4137) protect tubules by reducing oxidative stress [60,131,132,133].

The common nexus between disulfidptosis and H_2_S pathways is their targeting of thiol/disulfide chemistry [15]. In both, cysteine residues on proteins are modified: in disulfidptosis, they form extra disulfide bridges (–S–S–) linking actin filaments, whereas in H_2_S signaling, they are converted to persulfides (–S–SH). Both modifications are reversible by cellular reducing systems but have opposite functional consequences. In AKI, this nexus is critical: renal ischemia or toxin injury rapidly consumes NADPH and elevates ROS [60,134,135,136,137]. The resulting redox imbalance accelerates protein oxidation. In such stress, high H_2_S levels can shift the balance back by donating sulfide and boosting reductive capacity. Conversely, if H_2_S production falls (as observed in ischemic kidneys) or if NADPH is exhausted, disulfide formation predominates.

In AKI, proximal tubules often suffer mitochondrial dysfunction and NADPH drops, creating an environment like the cell-culture models that produce disulfidptosis. Indeed, histologically, AKI is characterized by actin disruption and brush border loss, possibly reflecting disulfide-linked collapse. Meanwhile, endogenous H_2_S (via CSE in tubules) normally defends these cells by maintaining glutathione and neutralizing oxidants [138,139,140,141,142]. H_2_S insufficiency, the “disulfide stress,” wins. This interplay suggests potential therapies: for example, boosting H_2_S or GSH might preserve NADPH pools and prevent disulfidptosis. Alternatively, controlling cystine uptake (SLC7A11 activity) or enhancing PPP flux could protect tubular thiols. AKI promotes conditions (NADPH depletion, ROS surge) that favor disulfidptosis, while H_2_S donors act to counteract this by preserving thiols and reducing glutathione levels and oxidative stress [60,140,141]. Thus, disulfidptosis and H_2_S biology converge on thiol/disulfide redox. A therapeutic window may exist wherein modulating H_2_S levels or redox cofactors could tip the scale away from cytoskeletal collapse toward cell survival. The roles of H_2_S in these mechanisms are shown in Table 2.

### 4.2. H_2_S as an Antagonist of Cytoskeletal Collapse

Disulfidptosis uniquely targets the actin cytoskeleton, causing it to collapse under disulfide crosslinking [15,19,36,37,49,50]. Intriguingly, H_2_S has been shown to positively regulate F-actin structure and cell morphology. For example, in endothelial cells, H_2_S stimulates Rac1 GTPase and PI3K pathways, leading to phosphorylation of cofilin and enhanced actin polymerization [144]. In experimental studies, H_2_S (via NaHS treatment) induced lamellipodia formation and reorganization of the actin cytoskeleton, effects that were abolished by Rac1 inhibition [144]. In other words, H_2_S triggers a Rac1–cofilin cascade that promotes actin network stability and protrusions [144]. By contrast, disulfidptosis produces fragmented actin fibers and loss of cell shape [15,19,36,37,49,50,144].

Thus, one can view H_2_S as an antagonist of disulfidptosis at the level of the cytoskeleton. By maintaining actin dynamics and preventing excessive F-actin aggregation, H_2_S may counter the collapse induced by disulfide bonding. It is possible that H_2_S-mediated sulfhydration of actin-regulatory proteins (like cofilin) keeps them in an active state, allowing actin turnover despite oxidative challenges [144]. Additionally, by buffering oxidative stress, H_2_S may prevent the formation of disulfide bonds on actin in the first place. In sum, through Rac1/cofilin signaling and redox modulation, H_2_S supports cytoskeletal integrity–precisely the structure targeted in disulfidptosis [15,19,36,37,49,50,144].

### 4.3. Mitochondrial and Metabolic Regulation of H_2_S

AKI is closely tied to mitochondrial dysfunction and metabolic stress. Disulfidptosis was first discovered under glucose starvation [14,42], a metabolic challenge. Although renal ischemia includes loss of both oxygen and glucose, it similarly disrupts energy metabolism and the pentose phosphate pathway, reducing NADPH. In this setting, mitochondrial resilience is crucial.

In mammals, the excretion of H_2_S varies across different systems [105]. Within the body, H_2_S is predominantly metabolized by mitochondria [145]. The mitochondrial enzyme SQOR utilizes H_2_S and, with the assistance of thiosulfate sulfurtransferase (TST) and Persulfide dioxygenase (ETHE1), converts it into thiosulfate. Reduced glutathione plays a crucial role in this process. Subsequently, thiosulfate undergoes further oxidation by thiosulfate reductase and sulfite oxidase (SUOX), ultimately forming sulfate, which is excreted by the kidneys, as illustrated in Figure 4. Oxygen is essential for this metabolic pathway [146,147].

Importantly, coenzyme Q (CoQ) is closely associated with these enzymes. Research has shown that the absence of CoQ leads to decreased expression of SQOR, TST, ETHE1, and SUOX [148]. In the early stages of CoQ deficiency, SQOR levels drop significantly, impairing H_2_S oxidation; however, supplementation with CoQ can restore H_2_S metabolism without affecting its production [149]. While SQOR activity and protein levels decline, the protein levels of TST, ETHE1, and SUOX in the H_2_S oxidation pathway increase in fibroblasts. It remains unclear whether this increase is a temporary compensatory response or inversely related to the reduction in SQOR [148]. Thus, investigating the effects of CoQ deficiency on H_2_S metabolic enzymes is important for understanding how modulation of H_2_S metabolic pathways may regulate H_2_S concentration and influence various signaling pathways in the body [86].

Under normal physiological conditions, when tissue production of H_2_S exceeds its metabolic utilization, an alternative metabolic pathway, cytoplasmic methyltransferase-mediated methylation, is engaged. Currently, the two known methyltransferases in humans are thiopurine methyltransferase (TPMT) and thiol methyltransferase (TMT). TPMT specifically methylates thiopurine compounds, while TMT targets aliphatic mercaptan substrates. Mass spectrometry has been used to directly monitor methyl sulfide formation, allowing assessment of H_2_S methylation and kinetic parameters [150]. Additionally, human methyltransferase-like protein 7B has been shown to catalyze the transfer of a methyl group from S-adenosine 1-methionine to H_2_S and various exogenous mercaptan small molecules, thereby facilitating H_2_S metabolism [86,150]. Besides methylation, H_2_S can also be eliminated by methemoglobin or by metallic and nonmetallic molecules such as oxidized glutathione [87].

On the other hand, H_2_S profoundly affects mitochondrial function in kidneys. At low concentrations, H_2_S can serve as an electron donor for the electron transport chain, but at higher doses it inhibits cytochrome c oxidase, inducing a reversible hypometabolic state [60]. Feng and colleagues showed that H_2_S donors can “suspend” mitochondrial respiration and limit O_2_ consumption, which protects tubules during ischemia [60,138,139]. This may seem paradoxical, but by slowing metabolism, H_2_S actually conserves ATP and reduces oxidative stress in the hypoxic renal medulla [60]. In addition, H_2_S sulfhydrates key mitochondrial proteins: notably, it activates the deacetylase Sirtuin 3 (SIRT3) by forming persulfide bonds on its critical cysteines [86]. SIRT3 deacetylates targets like the fusion protein Optic Atrophy 1 (OPA1), components of ATP synthase, and Superoxide Dismutase 2 (SOD2) [86]. These modifications preserve mitochondrial membrane potential, enhance ATP production, and bolster antioxidant defenses [86,151].

Mito-targeted H_2_S donors illustrate this effect dramatically. For instance, AP39 (a lipophilic triphenylphosphonium-conjugated donor) delivers H_2_S specifically to mitochondria. In rodent models of renal ischemia and transplantation, AP39 greatly outperformed nonspecific donors: a subnanomolar dose of AP39 improved graft survival and reduced tubular necrosis, whereas the same dose of slow-release GYY4137 had negligible effect [86]. AP39 preserved mitochondrial integrity and ATP levels during cold storage, reducing ROS burst and apoptosis [86]. In fact, AP39’s protective potency was reported to be on the order of 1000-fold greater than GYY4137 [86]. These findings underscore that bolstering mitochondrial function via targeted H_2_S can resist the energy collapse that leads to disulfidptosis.

In summary, H_2_S influences kidney cell metabolism in ways antithetical to disulfidptosis. By tempering mitochondrial respiration under stress [60], by activating mitochondrial quality controls (e.g., SIRT3/OPA1) [86], and by conserving ATP and NADPH, H_2_S donors create an environment less permissive to disulfide-driven death. Metabolically, H_2_S “rewires” ischemic cells toward survival.

### 4.4. Evidence of H_2_S Modulation in Kidney Injury Model

H_2_S plays multiple roles in the initiation and progression of kidney disease. AKI is a clinical syndrome characterized by a sudden decline in renal function, which can develop in individuals with or without preexisting kidney disease. The hallmark features of AKI include a rapid decrease in GFR, elevated serum creatinine levels, reduced urine output (oliguria), and in severe cases, complete absence of urine (anuria). AKI affects approximately 20% of hospitalized patients, but its incidence rises to 50% among those who are critically ill. The condition is classified into various types based on its underlying cause and stage, with prognosis and treatment strategies differing accordingly. Major complications of AKI include fluid overload, uremic complications, and disturbances in electrolyte balance. Globally, AKI is responsible for about 2 million deaths annually, imposing a significant burden on both healthcare systems and society [152,153]. Although disulfidptosis per se has not yet been demonstrated in animal AKI, numerous studies show H_2_S modulation markedly affects renal injury outcomes. These studies provide circumstantial support that H_2_S and redox pathways intersect in AKI.

#### 4.4.1. Role of H_2_S in Renal Ischemia/Reperfusion Injury

Ischemia–Reperfusion Injury (IRI) causes significant damage to microvascular structures and parenchymal organs, primarily through the generation of ROS, as demonstrated in various organs. The kidneys are especially susceptible to this form of injury, often resulting in AKI. ROS not only induce cell necrosis and apoptosis but also trigger lipid peroxidation and depletion of ATP [134,137]. Recent research has highlighted a strong link between H_2_S and renal IRI. For instance, Han et al. observed that after 30 min of bilateral renal ischemia in mice, both the expression and activity of CSE and CBS in the kidneys were reduced, leading to decreased H_2_S levels [132]. Administration of NaHS restored renal function and promoted normalization of tubular morphology. Additionally, NaHS treatment improved renal function by decreasing oxidative stress. Eelke et al. demonstrated that CSE contributes to renal protection during ischemia, likely by producing H_2_S to counteract oxidative stress [131]. Similarly, Azizi et al. reported that H_2_S protects against ischemia/reperfusion-induced AKI by reducing oxidative stress [133].

H_2_S may improve renal IRI through multiple pathways. Ischemia significantly impacts the renal endothelium, causing microvascular dysfunction that perpetuates inadequate blood flow and further renal damage [154]. One key mechanism involves H_2_S-induced vasodilation in renal smooth muscle tissue via activation of K^+^ ATP channels, which enhances renal blood flow and supports tubular recovery [65]. Additionally, the activity of the renin–angiotensin–aldosterone system (RAAS), a critical endocrine system controlling electrolyte balance, blood volume, and blood pressure [100,101,102], is regulated by renin release from juxtaglomerular (JG) cells, a process modulated by intracellular cAMP levels [61,98,99].

H_2_S has been found to reduce cAMP levels by inhibiting adenylyl cyclase (AC) across various cell types, implying a potential role in regulating renin release and decreasing Ang-II concentrations [61,155]. Laggner et al. demonstrated that H_2_S directly suppresses Angiotensin-Converting Enzyme (ACE) activity in a dose-dependent manner [103]. Beyond AC, recent studies indicate that ROS are also targeted by H_2_S, contributing to lowered renin levels in DN and suggesting the involvement of multiple mechanisms [61,94]. Importantly, H_2_S has been reported to alleviate renal stress in mice with Ang II-induced hypertension, whereas IRI can activate the RAAS pathway [156]. Collectively, these findings support the hypothesis that, through this second mechanism, H_2_S inhibits the RAAS system and preserves kidney function.

In the third mechanism, H_2_S preserves mitochondrial function. Administration of H_2_S donors can trigger a reversible hypometabolic state, primarily by temporarily reducing mitochondrial activity through reversible binding to cytochrome c oxidase. During hypoxia, mitochondrial integrity and function are compromised; however, this damage is mitigated following H_2_S treatment. As a result, H_2_S decreases oxygen consumption, shields tissues from hypoxia and shock, and offers protection against IRI [138,139].

The fourth mechanism involves the conversion of homocysteine to cysteine within cells, facilitating glutathione synthesis. H_2_S enhances homocysteine transport, boosts glutathione production, inhibits NADPH oxidase activity, scavenges free radicals, and increases antioxidant enzyme activity [140,141]. Furthermore, A39—a mitochondria-targeted H_2_S donor—has been shown to reduce cellular oxidative stress and provide dose-dependent protective effects against renal epithelial cell injury in vitro and IRI in vivo [157].

#### 4.4.2. Role of H_2_S in Drug-Induced AKI

Numerous nephrotoxic drugs are administered either alone or in combination with other medications. The mechanisms underlying drug-induced AKI are diverse but most often involve acute tubular or tubulointerstitial damage. Both drug-related and patient-specific factors contribute to increased risk [158]. Cisplatin, a chemotherapeutic agent commonly used to treat solid tumors [159], is associated with nephrotoxicity in approximately 25–30% of treated patients, manifesting frequently as AKI. Central to this injury is mitochondrial dysregulation: studies indicate that cisplatin accumulates within the mitochondria of renal proximal tubular cells, disrupting mitochondrial redox balance and ultimately leading to mitochondrial dysfunction [160,161].

Recent studies have shown that in mice with cisplatin-induced AKI, mitochondrial damage not only impairs cellular energy metabolism but also triggers mitochondrial fragmentation and excessive production of ROS, both of which contribute to the severity of tubular epithelial cell injury and death [107]. While earlier research has indicated that H_2_S can alleviate cisplatin-induced AKI, some contradictory findings have also been reported [162]. H_2_S increases the expression of SIRT3 and enhances its deacetylase activity by sulfhydrating SIRT3 at two CXXC zinc finger motifs. SIRT3, predominantly located in mitochondria, is a NAD^+^-dependent deacetylase and a key regulator of mitochondrial function. By mediating the deacetylation of its target proteins, SIRT3 improves mitochondrial bioenergetics and suppresses mitochondrial dysfunction [163]. Evidence suggests that H_2_S mitigates AKI in cisplatin-treated mice [28]. Moreover, recent studies have found that low concentrations of H_2_S can stimulate mitochondrial oxidative phosphorylation and reduce mitochondrial oxidant production [164]. Ahangarpour et al. also demonstrated that H_2_S can prevent the progression of cisplatin-induced nephrotoxicity in rats, likely due to its antioxidant effects [165].

Recent studies have also shown that H_2_S can worsen cisplatin-induced kidney damage. Francescato et al. demonstrated that inhibiting endogenous H_2_S production in rats with DL–propargylglycine (PAG) reduced inflammation and alleviated renal injury [166]. Similarly, Liu et al. observed that the slow-releasing H_2_S donor GYY4137 intensified cisplatin-induced kidney injury, which was linked to increased inflammation, oxidative stress, and apoptosis. Importantly, the dose of GYY4137 used in their study was relatively low, potentially resulting in insufficient or negligible H_2_S release [167]. Additionally, another study reported that in doxorubicin-induced renal damage, PAG provided protection by lowering H_2_S production and reducing both oxidative stress and inflammation in the kidneys [168].

It is clear from the studies that H_2_S plays a significant role in drug-induced AK. Nonetheless, some studies have reported conflicting results. These discrepancies may be due to variations in the concentrations of H_2_S used in different experiments, highlighting the need for further investigation and discussion.

#### 4.4.3. Role of H_2_S in Sepsis-Associated AKI

The likelihood of sepsis in critically ill patients with acute kidney injury (AKI) is around 40–50%. When sepsis and AKI develop concurrently, a condition known as sepsis-associated AKI (SA-AKI), mortality rates can reach up to 70% [169]. The primary cause of SA-AKI is kidney hypoperfusion. The underlying mechanisms of SA-AKI are complex, involving both macro- and microcirculatory disruptions in the kidneys, heightened inflammatory responses, increased oxidative stress, and activation of the coagulation cascade [170].

LPS, TNF-α, and interleukin-1β (IL-1β) are classic inflammatory cytokines that activate Toll-like receptor 4 (TLR4) signaling pathways and contribute to ROS production in mouse models of AKI [171]. Excessive ROS generation impairs tubular epithelial cell function. NaHS has been shown to improve renal function and reduce kidney histopathological alterations, LPS-induced inflammation, and oxidative stress. Chen et al. reported that plasma H_2_S levels decrease in rabbits with sepsis-associated AKI (SA-AKI) and that administration of exogenous H_2_S suppresses NF-κB and TNF-α activity while increasing IL-10 levels, thereby delaying kidney injury [172]. Similarly, Caitlyn et al. demonstrated that NF-κB transactivation drives inflammation and cellular hyperpermeability, but the CSE/H_2_S system can inhibit this process [173].

Unlike its established functions in other contexts, the role of H_2_S in sepsis remains uncertain. Elevated plasma H_2_S levels have been observed in patients experiencing septic shock [174]. In a mouse model of LPS-induced inflammation, LPS administration increases CSE gene expression in the kidneys. Pretreatment with PAG prior to LPS injection decreases both H_2_S content and leukocyte infiltration in renal tissue [60]. Additionally, administration of NaHS results in a significant rise in plasma TNF-α levels [128]. Contrarily, administration of NaHS attenuated the increase in plasma TNF-α and ICAM-1 levels in hepatic ischemia–reperfusion (HIR) rats [175,176]. Despite these findings, more studies are necessary to clarify the precise role of H_2_S in SA–AKI. Table 3 provides a summary of H_2_S involvement in AKI.

Taken together, the evidence suggests that H_2_S supplementation robustly protects the kidney across multiple AKI models [86]. This protective pattern—preserving redox status, maintaining the cytoskeleton, and sustaining mitochondria—is exactly what would counteract disulfidptotic triggers, i.e., NADPH loss and actin oxidation. Hence, while direct data on disulfidptosis in AKI are lacking, the published literature strongly implicates H_2_S-redox interplay in renal injury outcomes.

## 5. Therapeutic Opportunities of H_2_S

### 5.1. Effects of Fast and Slow Releasing H_2_S Donors in AKI

AKI is a serious, life-threatening disorder with multiple causes, including ischemic damage and exposure to nephrotoxic agents [179,180]. Due to the kidney-protective effects of H_2_S, pharmacologic H_2_S donors are of great therapeutic interest in AKI [86], and these donors can be broadly classified by their release kinetics and targeting.

Fast-releasing inorganic sulfides NaHS and Na_2_S release a bolus of H_2_S. These salts rapidly elevate H_2_S levels and have been widely used in preclinical AKI studies. In rodents, intravenous NaHS (50–100 μmol/kg) prior to IRI or cisplatin markedly improved renal function [86]. However, their rapid reactivity can cause transient hypotension, and their use is mainly experimental. The H_2_S donors, NaHS and GYY4137, have been shown to attenuate cisplatin-induced cell death and renal toxicity [181]. In diabetic rat kidneys, NaHS treatment decreased the expression of TGF-β1, Extracellular signal-regulated kinase 1/2 (ERK1/2), Tissue inhibitors of metalloproteinases (TIMPs), and Matrix metalloproteinases (MMPs), which contributed to the amelioration of renal fibrosis [182,183]. Renal fibrosis is linked to TGF-β/Smad signaling, AMP-activated protein kinase (AMPK) activation, ERK1/2 expression, and imbalances in MMP/TIMP regulation [182,183].

Slow-releasing donor compounds like GYY4137 release H_2_S gradually over hours. They produce lower peak H_2_S but more sustained levels. GYY4137 has demonstrated efficacy in reducing renal injury markers in animal models of AKI, e.g., improved survival in endotoxemia and cisplatin nephropathy [181]. H_2_S may enhance the recovery of renal function following obstruction and reduce kidney damage caused by unilateral ureteral obstruction (UUO). Evidence indicates that H_2_S could inhibit fibrosis by modulating the TGF-β1-driven EMT pathway. Collectively, these findings imply that H_2_S represents a promising new therapeutic option for improving renal function and minimizing kidney injury associated with obstructive uropathy [184].

Further research shows that reduced H_2_S levels lead to decreased miR-194 expression and increased collagen deposition and realignment, ultimately promoting fibrosis and renovascular constriction in diabetes. The H_2_S donor compound GYY4137 alleviates renal fibrosis in diabetes through a miR-194-dependent mechanism [185].

Endogenous H_2_S enhancers, such as certain drugs (e.g., S-propargyl-cysteine, taurine), can upregulate CBS/CSE or provide H_2_S substrates (e.g., L-cysteine) [186,187]. This indirect approach has shown benefits; for example, S-propargyl-cysteine increased H_2_S in IRI and reduced injury [186,187,188,189,190]. Natural compounds such as diallyl trisulfide from garlic also release H_2_S [191].

The mitochondrial-targeted H_2_S donor AP39 has been shown to significantly enhance the survival and function of donor kidneys following transplantation while also reducing cellular apoptosis and necrosis [157,192]. During cryopreservation of donor kidneys, H_2_S can mitigate apoptosis and necrosis, potentially improving post-transplant survival and function by regulating mitochondrial membrane potential and lowering ROS production [192]. Oxidative stress generated by glucose oxidase leads to mitochondrial dysfunction, depleting ATP in renal epithelial cells, increasing ROS levels, and promoting necrosis. Both in vitro and in vivo studies have demonstrated that pretreatment with AP39 confers a concentration-dependent protective effect against renal IRI [157]. The protective efficacy of AP39 as an H_2_S donor is approximately 1000 times greater than that of GYY4137, a nonspecific exogenous H_2_S donor [86,192]. These findings suggest that mitochondrial targeting optimizes H_2_S bioavailability precisely where it is needed most, within mitochondria during oxidative stress. Table 4 summarizes various H_2_S donors and their specific roles in acute kidney injury (AKI).

### 5.2. Targeting SLC7A11/Cystine Metabolism

Modulating SLC7A11 activity could reduce cystine-driven disulfide stress under metabolic impairment. Since disulfidptosis centers on cystine uptake by SLC7A11, modulating this pathway offers another strategy. In principle, reducing cystine imports would prevent intracellular disulfide overload at the cost of lowering glutathione. Modulators of the cystine/SLC7A11 pathway in renal cells are mentioned in Table 5.

#### 5.2.1. SLC7A11 Inhibitors

Agents like sulfasalazine, erastin, and sorafenib block system Xc^−^ (the cystine/glutamate antiporter) [37]. Sulfasalazine (an anti-inflammatory drug) and erastin are classic ferroptosis inducers that prevent cystine uptake. In kidney cells, blocking SLC7A11 would inhibit glutathione synthesis and ordinarily promote ferroptosis. However, in an AKI setting with high SLC7A11, partial inhibition could theoretically reduce the cystine-driven component of disulfidptosis [37,194,195].

#### 5.2.2. SLC7A11 Activators

Upregulating SLC7A11 can protect against ferroptosis by raising GSH. For example, the adipokine chemerin was found to activate AMPK/NRF2 signaling and increase SLC7A11 transcription in tubular cells, boosting cystine uptake and glutathione [196]. In a mouse I/R model, recombinant chemerin markedly attenuated AKI, an effect attributed to enhanced NRF2-SLC7A11-driven cysteine import. Agents like Nrf2 activators (sulforaphane, bardoxolone) or certain microRNAs (under investigation) could similarly upregulate SLC7A11. In the renal context, promoting SLC7A11 may be protective by preventing toxic lipid peroxidation; it might also paradoxically increase risk for disulfidptosis if metabolic stress occurs [196].

#### 5.2.3. Clinical Cystine Modulators

Cysteamine is used for cystinosis (lysosomal cystine storage); its role in cytosolic cystine and disulfidptosis is unclear. Similarly, one could use cystine-starvation diets or manipulation of homocysteine–cysteine balance to influence SLC7A11 flux. Remarkably, evidence shows that disturbing SLC7A11 function impacts AKI outcomes. The mycotoxin patulin was shown to inhibit SLC7A11 in renal cells via AMPK-Beclin1, provoking ferroptosis and worsening folic-acid-induced AKI [197]. Conversely, agents that upregulate SLC7A11 (like chemerin) protect against ischemic AKI. These findings imply that targeted modulation of cystine metabolism, either by blocking or boosting SLC7A11 depending on context, could be therapeutically exploited in AKI [196].

### 5.3. NADPH-Restoring Strategies

#### 5.3.1. Antioxidants/ROS Scavengers

Agents like Tempol (a superoxide dismutase mimetic) and Trolox (a vitamin E analog) can lower ROS and thereby reduce NADPH consumption. In fact, in the foundational disulfidptosis studies, adding Tempol or Trolox (or even 2-deoxyglucose, which shifts metabolism) completely rescued cells from actin collapse under glucose starvatio [14]. These compounds blunt the oxidative cascade, indirectly conserving NADPH.

#### 5.3.2. NADPH Oxidase Inhibitors

Drugs that block NADPH oxidase (NOX) enzymes (e.g., apocynin, gp91ds-tat peptide) would limit pathological ROS production in AKI. By curtailing NADPH usage by NOX, they help maintain reductive capacity. H_2_S itself has NOX-inhibitory effects [86], but combining H_2_S donors with dedicated NOX inhibitors could be synergistic.

#### 5.3.3. Pentose Phosphate Pathway (PPP) Activators

Stimulating PPP flux is an attractive idea, as this is the cell’s main source of NADPH. Pharmacologically, one might use ML337 (an experimental drug that acts as a negative allosteric modulator selective for the glutamate receptor mGluR3) or other Glucose-6-phosphate dehydrogenase (G6PD) activators to enhance NADPH generation [14,198]. Likewise, activating Nrf2 boosts expression of PPP enzymes and NADPH-producing isocitrate dehydrogenases [199].

#### 5.3.4. NADPH Donors

Direct NADPH supplementation is not clinically feasible, but some analogs (e.g., methylene blue) can act as redox shuttles to increase NADPH pools. Methylene blue has been studied in severe shock models to improve redox [200].

#### 5.3.5. H_2_S Donors

As noted, H_2_S conserves NADPH by inhibiting NOX and upregulating GSH synthesis [86]. Thus, using H_2_S donors, especially mitochondria-targeted ones like AP39, effectively restores redox balance and spares NADPH in AKI [86]. NADPH-restoring treatments aim to counter the metabolic trigger of disulfidptosis. Indeed, antioxidants like Tempol or metabolic modulators (2-DG) were shown to block F-actin oxidation in vitro.

### 5.4. Combination Approaches

Given the multifactorial nature of AKI and CKD, combination therapies targeting both disulfidptosis and other death pathways may be most effective. One promising concept is to pair H_2_S donors with ferroptosis or apoptosis inhibitors. For example, an H_2_S donor (e.g., AP39) plus a lipophilic antioxidant (ferrostatin-1) could concurrently block disulfide and lipid peroxidation damage. Alternatively, combining an xCT modulator with an NADPH-sparing agent might fine-tune cystine levels without depleting power. Small-molecule NRF2 activators to bolster both GSH and NADPH could be given together with H_2_S donors to amplify antioxidant defenses at multiple levels. To date, few studies have explicitly tested such combinations in AKI. However, the emerging insights suggest that synergistic strategies, such as H_2_S donor plus anti-inflammatory agent or H_2_S donor plus iron chelator, warrant investigation. Rigorous preclinical trials will be needed to identify optimal multi-modal regimens that protect the kidney by simultaneously preventing disulfide stress and other lethal insults.

Given their shared redox axis, combining H_2_S donors with metabolic/thiol-stabilizing agents may yield synergistic kidney protection. H_2_S donors, whether fast-release NaHS, slow-release GYY4137, or mitochondria-targeted AP39, directly reduce disulfides and enhance antioxidant signaling [86,118]. Some H_2_S donors and their actions are listed in Figure 5. Metabolic stabilizers such as thiol precursors or Nrf2 activators (e.g., N-acetylcysteine, dimethyl fumarate) sustain NADPH and GSH pools, further buffering disulfide stress. For instance, N-acetylcysteine (NAC) replenishes cysteine/GSH and blunts inflammation—it markedly improves renal function and reduces tubular damage in cisplatin-AKI [201]. Likewise, dimethyl fumarate activates Keap1–Nrf2/HO-1, boosting antioxidant capacity and attenuating ischemia–reperfusion kidney injury [202]. These complementary actions suggest a rational synergy: H_2_S donors tackle existing disulfide stress, while metabolic stabilizers reinforce the cell’s reductive metabolism and antioxidant defenses. Combining them could suppress disulfidptosis thresholds in AKI [86,202]. A list of candidate agents with dual H_2_S and metabolic modes of action in kidney injury models is shown in Table 6.

## 6. Conclusions

In conclusion, the emerging connection between disulfidptosis and H_2_S underscores the centrality of redox balance in AKI. By integrating metabolic therapies, e.g., glucose/NADPH modulators, with H_2_S biology, it may be possible to develop combination treatments that forestall multiple death pathways. Such approaches have the potential to transform AKI management by converting mechanistic insights into effective therapies. H_2_S stands out as a protective agent that counteracts many aspects of disulfidptosis. H_2_S boosts antioxidant capacity, maintains mitochondrial integrity, and preserves cytoskeletal dynamics. In models of AKI, H_2_S donors and strategies that elevate endogenous H_2_S consistently have been shown to improve outcomes. This inverse relationship, where H_2_S suppresses injury while disulfide stress promotes it, points to important therapeutic intersections. Specifically, therapies that raise H_2_S or mimic its effects could offset the NADPH drain and actin damage central to disulfidptosis. Further preclinical translational research is required to understand targeting modalities to maximize renoprotection without systemic toxicity. Table 7 summarizes therapeutic strategies inspired by these mechanisms, from H_2_S donors to redox modulators.

## 7. Future Perspectives

Disulfidptosis is a newly recognized form of regulated cell death marked by excessive cystine accumulation, depletion of NADPH, and abnormal disulfide bond formation in the actin cytoskeleton, ultimately leading to cytoskeletal collapse and cell death [209]. The metabolic and oxidative conditions in AKI, e.g., glucose deprivation, redox imbalance, and mitochondrial dysfunction, are highly relevant to disulfidptosis. H_2_S donors and strategies that elevate endogenous H_2_S have been consistently shown to improve outcomes of disulfidoptosis in kidneys. Thus, the exploration of disulfidptosis in renal pathophysiology represents a promising and largely untapped research frontier.

## Figures and Tables

**Figure 2:**
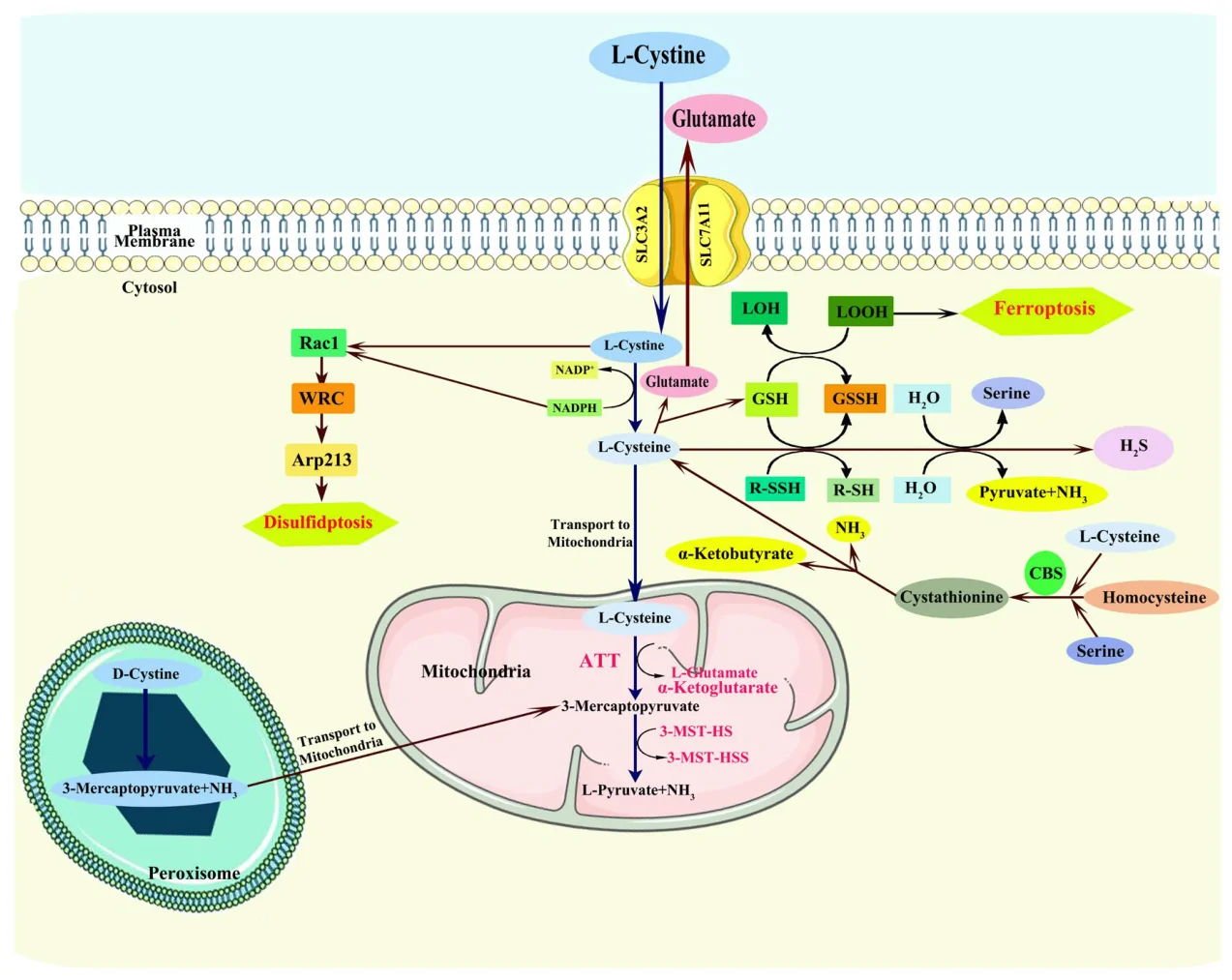
Interconnection of sulfide metabolism with disulfidptosis and ferroptosis signaling pathways. This diagram highlights cysteine’s pivotal role in intracellular sulfur metabolism, emphasizing its dual regulatory functions in H_2_S signaling, disulfidptosis, and ferroptosis. AST, aspartate aminotransferase; GSSH, Glutathione persulfide; LOH, Lipid alcohols; LOOH, Lipid hydroperoxides; SLC3A2, Solute carrier family 3 member 2.

**Figure 3:**
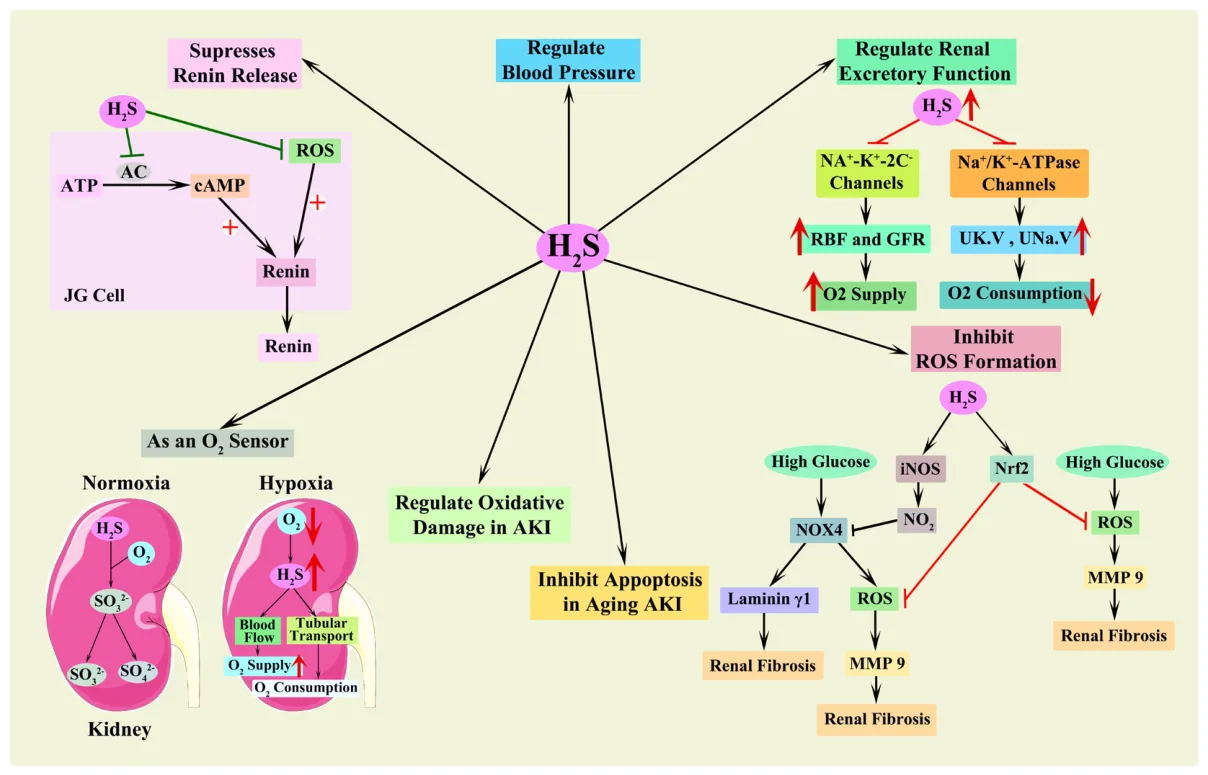
A multitude of effects of H_2_S on renal function. H_2_S inhibits the activity of tubular Na^+^-K^+^-2C^−^ channels (NKCC) and Na^+^/K^+^-ATPase (NKA), which enhances renal excretory functions such as glomerular filtration rate (GFR), urinary sodium excretion (UNa.V), and urinary potassium excretion (UK.V). H_2_S suppresses renin release in juxtaglomerular (JG) cells by inhibiting adenylate cyclase (AC) activity and reducing the production of ROS. H_2_S inhibits tubular Na^+^-K^+^-2Cl^−^ channels and Na^+^/K^+^-ATPase, thereby supporting renal excretory functions, including increased renal blood flow (RBF) and GFR, as well as restoration of oxygen levels. H_2_S acts as an oxygen sensor in the kidney. Under normoxic conditions, H_2_S is metabolized to sulfates in the presence of oxygen. During hypoxia (low O_2_), H_2_S accumulates, promoting the restoration of oxygen levels by increasing renal blood flow and decreasing tubular transport activity. Conversely, when H_2_S is deficient or at low physiological levels, as seen in aging or acute kidney injury (AKI), blood pressure, oxidative damage, inducible nitric oxide synthase (iNOS), and apoptosis increase, while heme oxygenase 1 decreases. Supplementation of H_2_S reverses these pathological changes. Black arrows indicate pathways, red upward arrows indicate upregulation, and ⊥ indicate blockade.

**Figure 4:**
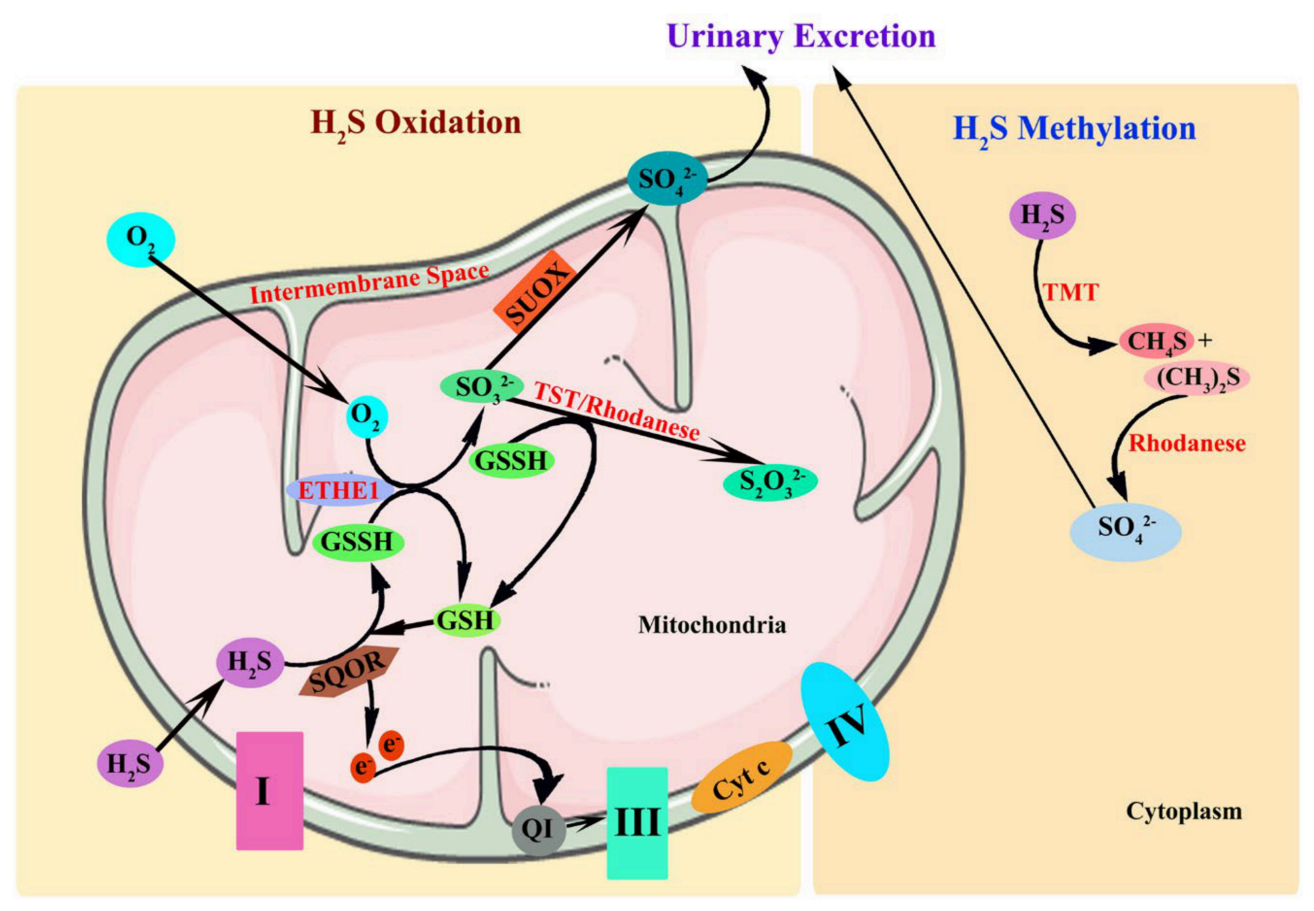
Model of mitochondrial metabolism of H_2_S: H_2_S is metabolized through two primary pathways: (1) oxidation in the mitochondria and (2) methylation in the cytoplasm. Among these, mitochondrial oxidation is considered the main route for H_2_S clearance. In this process, H_2_S is first oxidized by sulfide:quinone oxidoreductase (SQOR) to produce a persulfide intermediate. This persulfide is then further oxidized by ETHE1, resulting in the formation of sulfite (SO_3_^2−^). Sulfite is subsequently converted into sulfate (SO_4_^2−^) by sulfite oxidase and into thiosulfate (S_2_O_3_^2−^) by rhodanese. Both sulfate and thiosulfate are ultimately excreted in the urine, eliminating H_2_S from the body. The electrons released during H_2_S oxidation by SQR are transferred to ubiquinone and subsequently enter the electron transport chain at complex III. In the methylation pathway, H_2_S is transformed into methanethiol (CH_4_S) and dimethyl sulfide (CH_3_)_2_S through the action of S-methyltransferase (TMT). Dimethyl sulfide can also be further metabolized by rhodanese to generate sulfate, which is then excreted in the urine. Cyt c refers to cytochrome c.

**Figure 5:**
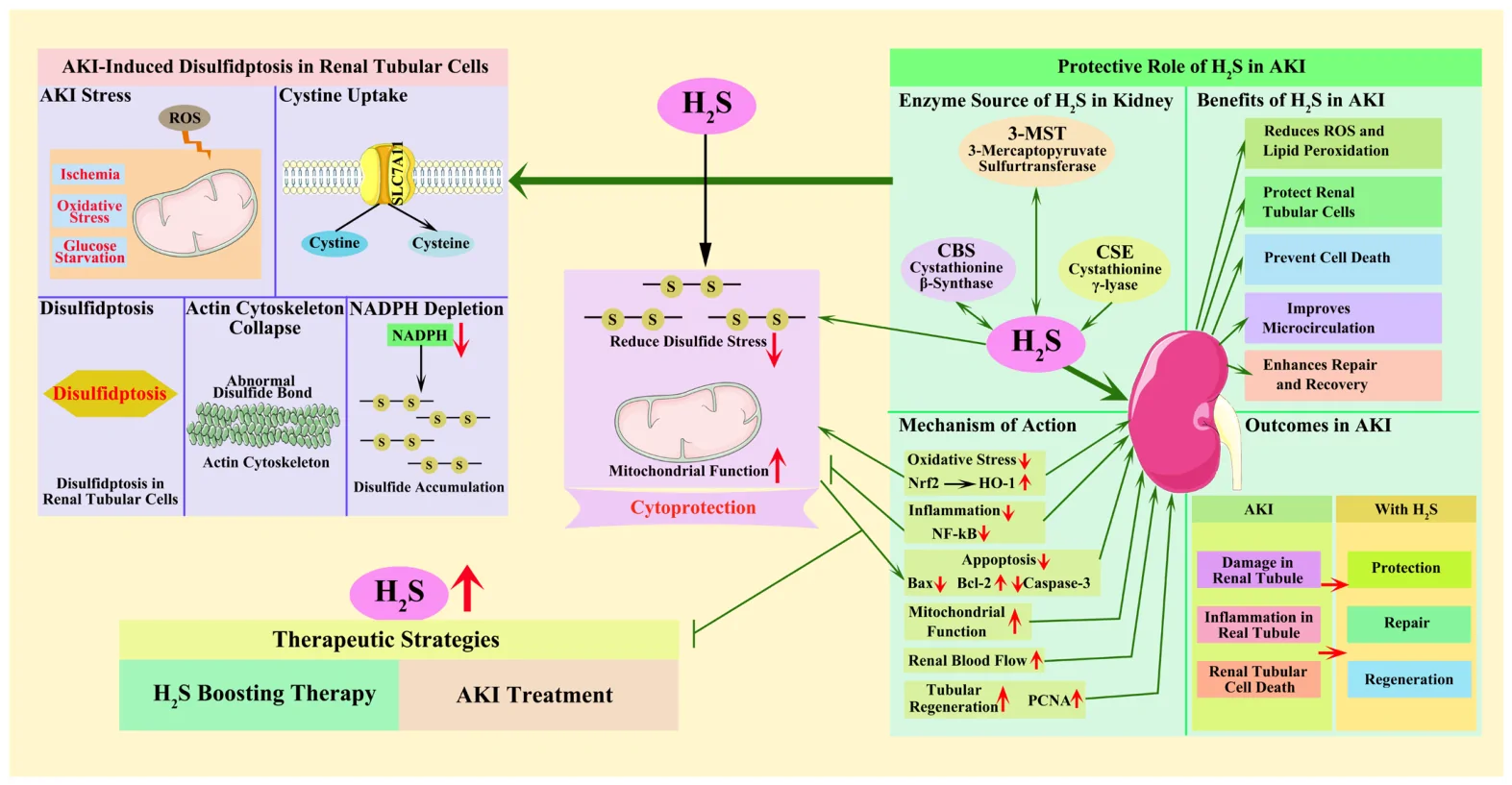
Interactions of disulfidptosis and H_2_S biology with AKI mechanisms. Green and black arrows indicate effects, products or pathways, red upward arrows indicate upregulation and downward arrows indicate downregulation, bidirectional arrows indicate two-ways movement, and ⊥ indicate blockade.

**Table 2:** Redox imbalance in disulfidptosis and role of H_2_S. → Indicates a mechanistic consequence, progression, or relationship leading from one event to another, and ↑ Indicates an increase, elevation, or upregulation.

Feature	Disulfidptosis	H_2_S	References
Redox trigger	NADPH depletion → ↑ disulfide stress	Sulfur donation (from H_2_S) → S-sulfhydration of protein thiols; antioxidant effects (↑GSH, ↑antioxidant enzymes)	[34,60,64]
Cytoskeletal effect	Actin disulfide crosslinking (F-actin contracts) → collapse	Actin stabilization (persulfidation) → enhanced polymerization	[64,126,143]
Implications in AKI	Tubular injury/cell death via disulfide stress (e.g., glucose- or ischemia-induced)	Tubular protection: dampens ROS and preserves redox balance (e.g., mitigates I/R injury)	[34,60,126,132]

**Table 3:** Experimental evidence of H_2_S modulation in AKI models. ↑ = increased/elevated/upregulated, and ↓ = decreased/reduced/downregulated.

Injury Model	Intervention	Effects on Kidney	Key Findings	References
Ischemia–Reperfusion	CSE/CBS inhibitors (e.g., PAG)	↓ H_2_S, ↑ tubular necrosis, inflammation	Inhibitors aggravated AKI; pro-H_2_S signaling protective	[177]
	NaHS or Na_2_S donor	↑ blood flow/GFR, ↓ oxidative stress, ↓ apoptosis	Donors improved function and reduced ROS	[86]
Cisplatin toxicity	NaHS, GYY4137 donors	↓ tubular apoptosis, ↑ GSH/Nrf2, stabilized mitochondria	Donors attenuated nephrotoxicity via SIRT3 and Nrf2	[167,177]
Gentamicin nephropathy	NaHS donor	↓ TNF-α/NO, ↑ HO-1/IL-10, ↓ iNOS	Anti-inflammatory, antioxidant effects	[178]
Septic AKI (LPS)	NaHS donor	↑ autophagy, ↓ inflammation	(various studies) H_2_S improved survival; mechanisms Nrf2/PKC/NLRP3	[86]
Transplant/Storage	AP39 (mito-targeted H_2_S)	↑ graft viability, ↓ necrosis during cold storage	AP39 (1000x potency) preserved mitochondria	[157,177]

**Table 4:** Representative H_2_S donors and their role in AKI. ↑ = increased/elevated/upregulated.

Donor	Type/Release	Targeting	Notes in AKI Context	Reference
NaHS/Na_2_S	Fast-releasing	Systemic	Rapid ↑[H_2_S], used i.v. to attenuate IRI and toxicity	[178,181,193]
GYY4137	Slow-releasing	Systemic	Sustained low [H_2_S]; improved outcome in AKI models	[86,181]
AP39	Mito-targeted	Mitochondria	Profound protection in IRI and transplant models (10^3^× potency vs. GYY4137)	[86,192]
AP123	Mito-targeted	Mitochondria	Inhibition of hyperglycemia-induced oxidative stress and metabolic alterations in microvascular endothelial cells in vitro	[164]

**Table 5:** Modulators of cystine/SLC7A11 pathway in renal cells. ↑ = increased/elevated/upregulated, ↓ = decreased/reduced/downregulated.

Modulator	Action on SLC7A11/Cystine	Renal Effects	Reference
Sulfasalazine	SLC7A11 inhibitor	Known ferroptosis inducer (used in vitro)	[15,37,194,195]
Erastin	SLC7A11 inhibitor	Induces ferroptosis; potential to reduce cystine uptake	[37,194,195]
Glutamate	Competitive inhibitor (xCT)	Blocks cystine import, mimics erastin effect	[37,194,195]
Chemerin (C9)	Indirect activator (via AMPK/NRF2)	↑ SLC7A11 expression, ↑ cystine/GSH, ↓ AKI severity	[50,196]
Patulin (toxin)	SLC7A11 inhibitor	↓ SLC7A11 activity, ↑ ferroptosis, worsens AKI	[197]
Nrf2 activators	↑ NRF2→↑ SLC7A11 transcription	Potentially ↑ GSH, counter lipid-ROS	[196]

Targeting cystine transport must balance antioxidant defenses and disulfide load. Preclinical data shows both blockade (e.g., by patulin) and upregulation (e.g., via NRF2) of SLC7A11 can markedly alter AKI outcomes [196,197].

**Table 6:** Representative candidate agents with dual H_2_S and metabolic modes of action that have shown renal protective effects in kidney injury models.

Candidate Agent	Mechanism & Target	Evidence in AKI Models (with Citation)	Reference
NaHS (fast H_2_S donor)	Releases H_2_S to reduce protein disulfides; inhibits inflammation/ oxidative stress	Improves renal function and histology in LPS-AKI; suppresses TLR4/NLRP3-mediated inflammation and lipid peroxidation	[107]
GYY4137 (slow H_2_S donor)	Sustained H_2_S release; activates Nrf2 signaling	Reduces cisplatin-induced renal cell death/toxicity (also improves renal blood flow and GFR in obstructive nephropathy models)	[86,107]
AP39 (mito-targeted H_2_S)	Targets H_2_S to mitochondria; limits ROS/inflammation	Dose-dependently protects against renal ischemia–reperfusion: lowers BUN/Cr, MPO and MDA, and apoptosis in rats	[157]
NAC (thiol donor)	GSH precursor; restores cysteine thiol pools; anti-inflammatory	Attenuates cisplatin nephrotoxicity: improves BUN/Cr and reduces tubular damage, inflammation and oxidative stress	[201]
Dimethyl fumarate (metabolic stabilizer)	Activates Nrf2/HO-1; boosts antioxidant enzymes	Mitigates renal IRI by reducing inflammation and lipid peroxidation via Nrf2/HO-1/NQO1 pathway activation	[202]

Each of these agents has an antioxidant/anti-inflammatory footprint that opposes disulfide stress. Future studies should test such combinations (e.g., NaHS + NAC or AP39 + DMF) in AKI models to determine if they synergize in preserving thiol homeostasis and preventing disulfidptosis-driven injury [107,201].

**Table 7:** Summary of therapeutic strategies targeting disulfidptosis and redox in AKI. ↑ = increased/elevated/upregulated, ↓ = decreased/reduced/downregulated.

Strategy	Target/Mechanism	Expected Effect in AKI	Reference
H_2_S Donors (NaHS, AP39, etc)	↑ H_2_S; sulfhydration of proteins; ↓ ROS	Preserve mitochondria, cytoskeleton, GSH/NADPH; ↓ inflammation and apoptosis	[203]
SLC7A11 Modulation	Block or enhance cystine uptake (e.g., erastin or NRF2 activators)	Reduce disulfide load OR ↑ GSH (depending on approach); may suppress ferroptosis or disulfidptosis	[204,205]
Antioxidants (Tempol, Trolox)	Scavenge ROS, spare NADPH	Prevent actin oxidation (block disulfidptosis); protect tubules from ROS-induced injury	[14]
NADPH Oxidase Inhibitors	Inhibit NOX-mediated ROS production	Conserve NADPH; attenuate oxidative tubular damage	[206]
PPP/Nrf2 Activators	↑ NADPH generation; ↑ GSH synthesis	Counteract metabolic stress; bolster antioxidant defenses	[207]
Iron Chelators/Ferroptosis inhibitors	↓ lipid peroxides, ↑ GSH	Complement H_2_S effects by blocking alternate death pathways	[208]

## Data Availability

No new data were created or analyzed in this study. Data sharing is not applicable to this article.

## Abbreviations

The following abbreviations are used in this manuscript:

(CH_3_)_2_S	Dimethyl sulfide
2ME	2-mercaptoethanol
3-MP	3-mercaptopyruvate
3-MST	3-mercaptopyruvate sulfurtransferase
3-MST-HS	3-Mercaptopyruvate Sulfurtransferase-Hydrogen Sulfide
AC	Adenyl cyclase
ACE	Angiotensin-Converting Enzyme
ACPs	Actin cytoskeletal proteins
AKI	Acute Kidney Injury
Arp	Actin-related protein
AST	aspartate aminotransferase
ATP	Adenosine triphosphate
cAMP	Cyclic Adenosine Monophosphate.
CAT	Cysteine aminotransferase
CBS	Cystathionine-β-synthase
CH_4_S	Methanethiol
CSE	Cystathionine-γ-lyase
CYFIP1	Cytoplasmic FMR1-interacting protein 1
DAO	D-amino acid oxidase
DTT	Dithiothreitol
ERK1/2	Extracellular signal-regulated kinase ½
GFR	Glomerular filtration rate
GPX4	Glutathione peroxidase 4
GSH	Glutathione
H_2_S	Hydrogen sulfide
HO-1	Heme Oxygenase-1
iNOS	inducible nitric oxide synthase
IRI	Ischemia–Reperfusion Injury
LC3-II	Microtubule-Associated Protein 1 Light Chain 3-II
LOH	Lipid alcohols
LOOH	Lipid hydroperoxides
LPS	Lipopolysaccharide
MMP9	Matrix Metalloproteinase-9
NAC	N-acetylcysteine
NADK	Nicotinamide Adenine Dinucleotide Kinase
NADPH	Reduced nicotinamide adenine dinucleotide phosphate
NADPH	Reduced nicotinamide adenine dinucleotide phosphate
NAK	Na^+^/K^+^-ATPase channels
NCKAP1	NCK-associated protein 1
NKCC	Na^+^-K^+^-2C^−^ channels
NO_2_-	Nitrogen dioxide
NOX	NADPH oxidase
NQO1	NAD(P)H:quinone oxidoreductase 1
OPA1	Optic Atrophy 1
PPP	Pentose phosphate pathway
PTP1B	Protein Tyrosine Phosphatase 1B
Rac1	Ras-related C3 botulinum toxin substrate 1
RBF	Renal blood flow
ROS	Reactive oxygen species
R-SH	Thiol or sulfhydryl group
S_2_O_3_^2−^	Thiosulfate ions
SIRT3	Sirtuin 3
SLC3A2	Solute carrier family 3 member 2
SLC7A11	Solute carrier family 7 member 11
SO_3_^2−^	Sulfite ions
SO_3_^2−^	Sulfite ions
SO_4_^2−^	Sulfate ions
SO_4_^2−^	Sulfate ions
SOD2	Superoxide Dismutase 2
SQOR	Sulfide:quinone oxidoreductase
SUOX	Sulfite oxidase enzyme
TIMPs	Tissue inhibitors of metalloproteinases
TMT	Thiol S-methyltransferase
TST	Thiosulfate sulfurtransferase
Uk	V- Urinary potassium
Una	V-Urinary sodium
WRC	WAVE regulatory complex
